# Study of prevalence, risk factors for acute kidney injury, and mortality in liver cirrhosis patients

**DOI:** 10.1007/s11845-024-03663-z

**Published:** 2024-03-22

**Authors:** Pooja Basthi Mohan, Shankar Prasad Nagaraju, Balaji Musunuri, Siddheesh Rajpurohit, Ganesh Bhat, Shiran Shetty

**Affiliations:** 1 Department of Gastroenterology and Hepatology, Kasturba Medical College, Manipal Academy of Higher Education, Manipal, India; 2 Department of Nephrology, Kasturba Medical College, Manipal Academy of Higher Education, Manipal, India

**Keywords:** Acute kidney injury, Acute tubular necrosis, Hepatorenal syndrome, International Club of Ascites, Risk factors

## Abstract

**Introduction:**

Acute kidney injury (AKI) occurs frequently in patients with end-stage liver disease and cirrhosis and is associated with increased short-term mortality. This study aims to study the prevalence and risk factors associated with AKI development and mortality in cirrhosis of liver patients.

**Methodology:**

In the current prospective study, hospitalized patients with liver cirrhosis from October 2021 to March 2023 were recruited. Demographic, clinical, and laboratory data were collected, which included, the etiology of cirrhosis, comorbidities, severity of liver disease, and relevant biochemical parameters. The patient was followed up for 90 days to record the clinical outcome. The statistical software SPSS was utilized to conduct the analysis.

**Results:**

Of 364 liver cirrhosis patients, 25.2% (*n*, 92) had AKI and belonged to an average age of 51.54 ± 11.82 years. The majority of individuals in the study were males (90.4%), and alcohol (63.4%) was the most common etiology of liver cirrhosis. The present study showed that higher level of direct bilirubin (*p* = 0.011) and MELD score (*p* = 0.0001) were identified as significant risk factors for AKI development in patients with liver cirrhosis. Regarding mortality, the significant risk factors were the presence of AKI (*p* = 0.045) and MELD score (*p* = 0.025). Among AKI patients, 90-day mortality rates were higher in patients with acute tubular necrosis (*p* value = 0.010) and stage 3 AKI (*p* value = 0.001).

**Conclusion:**

AKI is common in cirrhosis of liver patients. Elevated levels of direct bilirubin and MELD score emerged as significant factors associated with AKI development. Furthermore, AKI and MELD scores were identified as independent risk factors for mortality at both 30 and 90 days. Survival rates were influenced by both the type and stage of AKI; AKI stage 3 and ATN patients had significantly higher mortality rate. Early AKI detection and management are crucial for reducing mortality risk in liver cirrhosis patients.

## Introduction

Acute kidney injury (AKI) is one of the most unfavorable consequences of liver cirrhosis, particularly in decompensated cirrhosis [1]. Among patients hospitalized with liver cirrhosis, a significant number suffer from AKI, while approximately 1% concurrently have chronic kidney disease (CKD) [2, 3]. AKI is one of the key predictors of short-term mortality in individuals with liver cirrhosis.

The prevalence of AKI in liver cirrhosis is ranging from 20 to 50% [4, 5]. Research conducted in India by Arora et al. [6] found a prevalence of 40.6% of AKI among individuals with liver cirrhosis. Serum creatinine (SCr) and urine output, two indicators of kidney function, are presently used to diagnose AKI [7–9].

AKI in liver cirrhosis has been redefined by the International Club of Ascites (ICA) as rise in sCr of at least 50% from baseline or an increase of at least 0.3 mg/dL in at least 48 h or at least 1.5 times baseline that has occurred within the previous 7 days. Furthermore, AKI has been classified into three stages (1–3) based on the magnitude of SCr elevation according to the ICA criteria. This staging system demonstrates a strong correlation with the prognosis of individuals with cirrhosis [9–11].

AKI can occur due to prerenal, intrinsic renal, and postrenal factors. Prerenal AKI (PRA) is the most frequent cause of AKI in hospitalized patients with liver cirrhosis, followed by hepatorenal syndrome (HRS) and acute tubular necrosis (ATN) [12, 13]. PRA represents the functional renal component whereas ATN involves structural damage to the kidney and is characterized by alterations in renal tubular cells [14].

HRS is an extreme form of functional kidney injury [15]; it is due to diminished renal blood flow, which are unresponsive to an increase in volume. Postrenal AKI in patients with liver cirrhosis is quite rare [13, 16, 17]. Accurate diagnosis of the causes of acute impairment of kidney function in cirrhosis is important as treatment and outcome vary significantly.

Managing patients with liver cirrhosis, especially with a focus on complications like AKI, requires a comprehensive and multidisciplinary approach. Early identification of risk and precipitating factors may be the guiding light for managing AKI, further improving overall clinical outcomes. The present study was planned to determine the prevalence, risk factors associated with the development of AKI and short-term mortality in liver cirrhosis patients, and the impact of AKI on the survival of cirrhosis patients.

## Methodology

This prospective study was conducted on patients with liver cirrhosis who were enrolled in the tertiary care center in Manipal, India, between October 2021 and March 2023. Patients with a confirmed diagnosis of liver cirrhosis and AKI and above the age of 18 years were included in the study after attaining the institutional ethical approval (IEC 338–2021) and CTRI registration (2021/09/036171). Liver cirrhosis was diagnosed based on biochemical, clinical, radiologic, or histopathologic evidence with F4 changes (wherever available). Diagnosis of the AKI was made based on ICA criteria, i.e., rise in sCr above 0.3 mg/dL within 2 days or ≥ 50% rise from baseline (within the previous 7 days) [9, 10].

### AKI staging

- ICA-AKI stage 1: increase in serum creatinine by 0.3 mg/dL or increase in serum creatinine by 50 to 100% from baseline
- ICA-AKI stage 2: increase in serum creatinine by 100 to 200% from baseline
- ICA-AKI stage 3: increase in serum creatinine by > 200% from baseline or increase in serum creatinine to 4 mg/dL with an acute increase by 0.3 mg/dL or need for renal replacement therapy [10]

Participants were also classified as having one of the three types of AKI: (1) PRA, (2) HRS, and (3) ATN.

- PRA: Patients with pre-renal state (such as bleeding or loss of gastrointestinal fluid) and improved sCr following the administration of volume and withdrawal of diuretics [18].
- HRS: HRS based on 2015 International Club Ascites Criteria for AKI [8–10].
- ATN: Participants failed to meet criteria for PRA and HRS with a clinical history consistent with tubular/parenchymal kidney injury with or without urinalysis that shows muddy brown granular casts [18, 19].

Demographic information, etiology, laboratory investigations, and decompensation events were documented using a predefined form. The severity of liver disease was evaluated using the Child–Turcotte–Pugh (CTP) and Model for End-stage Liver Disease (MELD) scores. Patients with CKD or who underwent liver or kidney transplants and lost follow-up will not be included in the study. All recruited patients were managed as per standard protocols and were followed up for 90 days.

### Statistical analysis

Mean ± SD represents continuous variables, whereas median with interquartile range is used for non-continuous variables. Percentages of categorical variables were compared using chi-square or Fisher exact test. We used *t*-test to compare the mean for continuous variables. We analyzed survival rates using Kaplan–Meier analysis. Univariate and multivariate regression analyses were used to assess the risk factors contributing for AKI development and mortality. A *p* value of < 0.05 was considered significant. Data were analyzed using SPSS version 20.0.

## Results

### Demographics and disease characteristics

Of 364 patients, 25.2% (n, 92) were AKI with most being male patients 90.4%. The participant’s mean age was 51.54 ± 11.82 years. Alcohol (63.3%) was the most commonly reported etiology followed by NASH (27.15%).

Patients with AKI showed a significant (*p* value: < 0.05) increase in jaundice, edema, ascites, and icterus compared to the non-AKI group. Liver cirrhosis patients in AKI showed significant increase in the following biochemical parameters, like TLC (10.92 ± 6.31 vs. 7.868 ± 6.66) total bilirubin (6.022 ± 5.30 vs. 3.71 ± 3.63), direct bilirubin (3.52 ± 2.96 vs. 2.01 ± 1.92), creatinine (2.022 ± 0.791 vs. 0.814 ± 0.17), urea (56.88 ± 27.87 vs. 23.06 ± 13.293), and INR (1.619 ± 0.398 vs. 1.42 ± 0.429) with significant decrease in serum albumin (2.57 ± 0.55 vs. 2.93 ± 0.763), and sodium (129.96 ± 5.84 vs. 132.21 ± 6.19) (Table 1).

**Table 1:** Demographics and disease characteristics

Variables	Liver cirrhosis with AKI (*n* = 272)	Liver cirrhosis without AKI (*n* = 92)	*p* value
Age	51.85 ± 11.507	50.95 ± 12.024	0.518
Etiology (%)			
Alcohol	63.80%	62.80%	
NAFLD	26.60%	27.70%	0.913
HBV	9.60%	9.60%	
Co-morbidity			
No co-morbidity	58 (61.7)	62 (66.0)	
1 co-morbidity	22 (23.4)	26 (27.7)	
2 co-morbidity	11 (11.7)	6 (6.4)	
≥ 3 co-morbidity	3 (3.2)	0 (0)	0.176
**Cirrhosis complication**			
Ascites	91 (96.8)	77 (81.9)	0.001*
Jaundice	52 (55.3)	35 (37.2)	0.013*
Edema	60 (63.8)	45 (47.9)	0.028*
Icterus	52 (55.3)	28 (29.8)	< 0.05*
Malena	14 (14.9)	13 (13.8)	0.835
**Disease severity score**			
CTP	6.24 ± 3.13	4.30 ± 3.70	< 0.05*
MELD	23.44 ± 6.50	17.94 ± 6.61	< 0.05*
**Complete blood count (mean ± SD)**			
Hemoglobin (g/dL)	9.3413 ± 1.90	10.45 ± 2.66	0.001*
HCT (%)	27.613 ± 5.46	30.45 ± 6.97	0.002*
Platelet (× 10^3^/µL)	118.18 ± 80.79	109.02 ± 52.397	0.359
TLC (× 10^3^/µL)	10.92 ± 6.31	7.868 ± 6.66	0.002*
**Liver functioning test**			
Total bilirubin (mg/dL)	6.022 ± 5.30	3.71 ± 3.63	0.001*
Direct bilirubin (mg/dL)	3.52 ± 2.96	2.01 ± 1.92	< 0.05*
Total protein (g/dL)	6.60 ± 2.061	6.522 ± 0.748	0.715
ALP (U/L)	133.67 ± 52.16	131.06 ± 66.78	0.767
ALT (IU/L)	65.19 ± 46.5	39.49 ± 24.56	0.326
AST (IU/L)	81.787 ± 56.57	82.78 ± 52.13	0.909
Globulin (g/dL)	3.85 ± 0.885	3.59 ± 0.767	0.32
Albumin (g/dL)	2.57 ± 0.55	2.93 ± 0.763	< 0.05*
**RFT and serum electrolytes**			
Potassium (mmol/L)	4.61 ± 0.92	4.225 ± 0.624	0.001*
Sodium (mmol/L)	129.96 ± 5.84	132.21 ± 6.19	0.012*
Creatinine (mg/dL)	2.022 ± 0.791	0.814 ± 0.17	< 0.05*
Urea (mg/dL)	56.88 ± 27.87	23.06 ± 13.293	< 0.05*
**Coagulation test**			
INR	1.619 ± 0.398	1.42 ± 0.429	0.002*
Prothrombin time	17.98 ± 4.709	15.44 ± 4.60	< 0.05*

*ALP* alkaline phosphatase, *ALT* alanine transaminase, *AST* aspartate aminotransferase, *CTP* Child–Turcotte–Pugh, *INR* international normalized ratio, *MELD* Model for End-stage Liver Disease, *RFT* renal function test, *TLC* total leukocyte count

^*^Significant

### AKI staging and classification

AKI staging was carried out as per the ICA-AKI criteria [10]. Out of 92 participants, 51.0% (*n* = 47) had AKI stage 1, 32.6% (*n* = 30) had AKI stage 2, and 16.3% (*n* = 15) had AKI stage 3.

In terms of AKI classification, hepatorenal syndrome was present in 42.39% (*n* = 39) of cases, while PRA was identified in 35.86% (*n* = 33), and ATN was observed in 21.73% (*n* = 20) of patients.

### Risk factors: development of AKI

A logistic regression was performed on the blood and biochemical parameters. Univariate analysis showed higher direct bilirubin, MELD, and CTP score as significant factors associated with the development of AKI in patients with liver cirrhosis. However, multivariate regression analysis revealed higher direct bilirubin [1.32 (1.069–1.653)] and MELD score [0.748 (0.727–0.845)] as significant factors associated with the development of AKI in patients with liver cirrhosis. The data were presented as odds ratios and 95% confidence intervals.

### Mortality

**Fig. 1:**
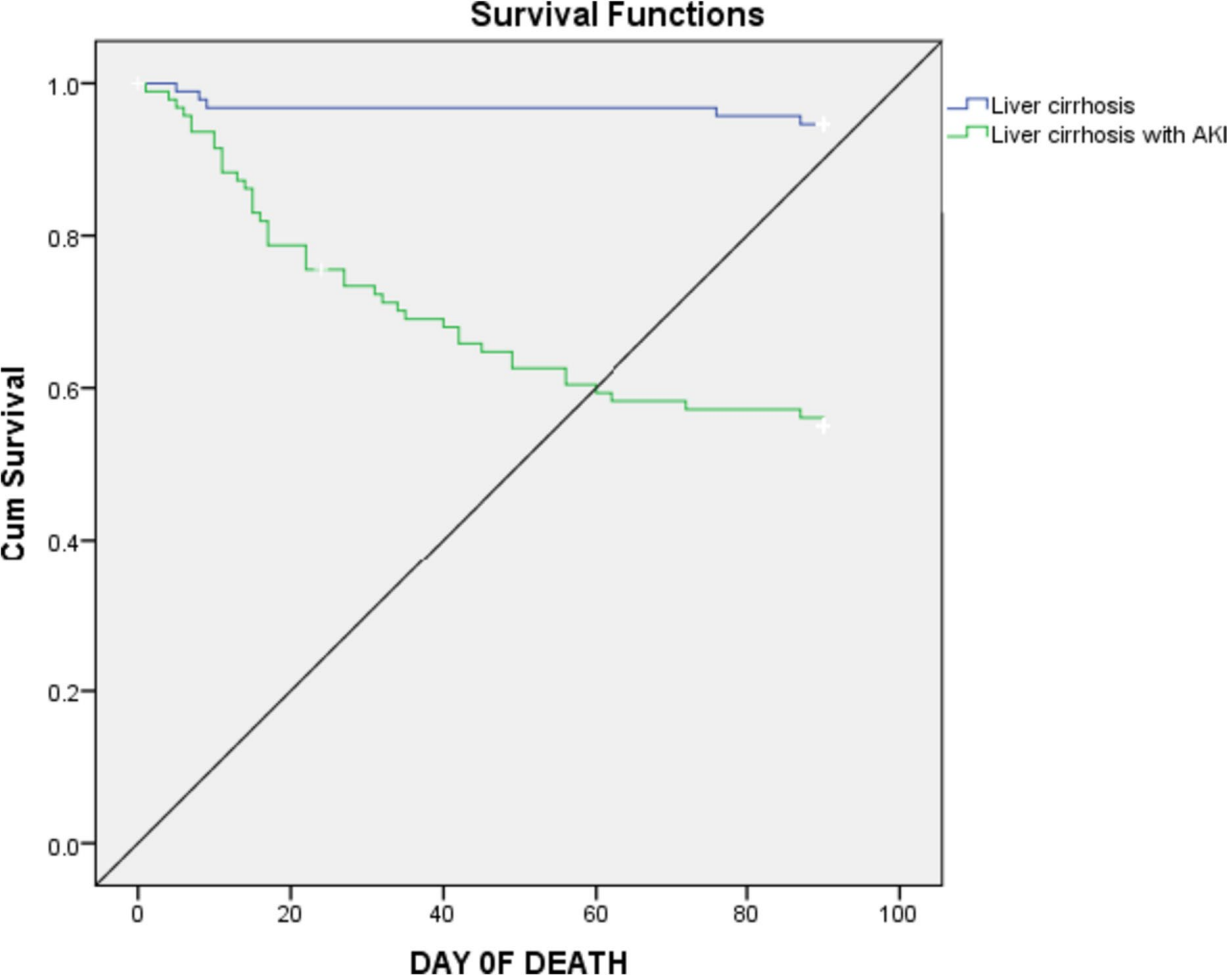
Kaplan–Meier survival curve showing 90-day survival in liver cirrhosis patients with and without AKI

The over-all in-hospital mortality rate was 4.37% (*n* = 16), while 30- and 90-day mortality rates were 14.01% (*n* = 51) and 23.35% (*n* = 85), respectively. The Kaplan–Meier survival analysis revealed that 90-day mortality was higher among liver cirrhosis patients with AKI (Fig. 1).

### Predictors of mortality

Multivariate regression analysis revealed presence of AKI [3.455 (1.028–11.611)] and MELD score [1.063 (1.005–1.124)] were independent risk factors for mortality in the study population. The data were presented as odds ratios and 95% confidence intervals.

### Mortality and AKI types

**Fig. 2:**
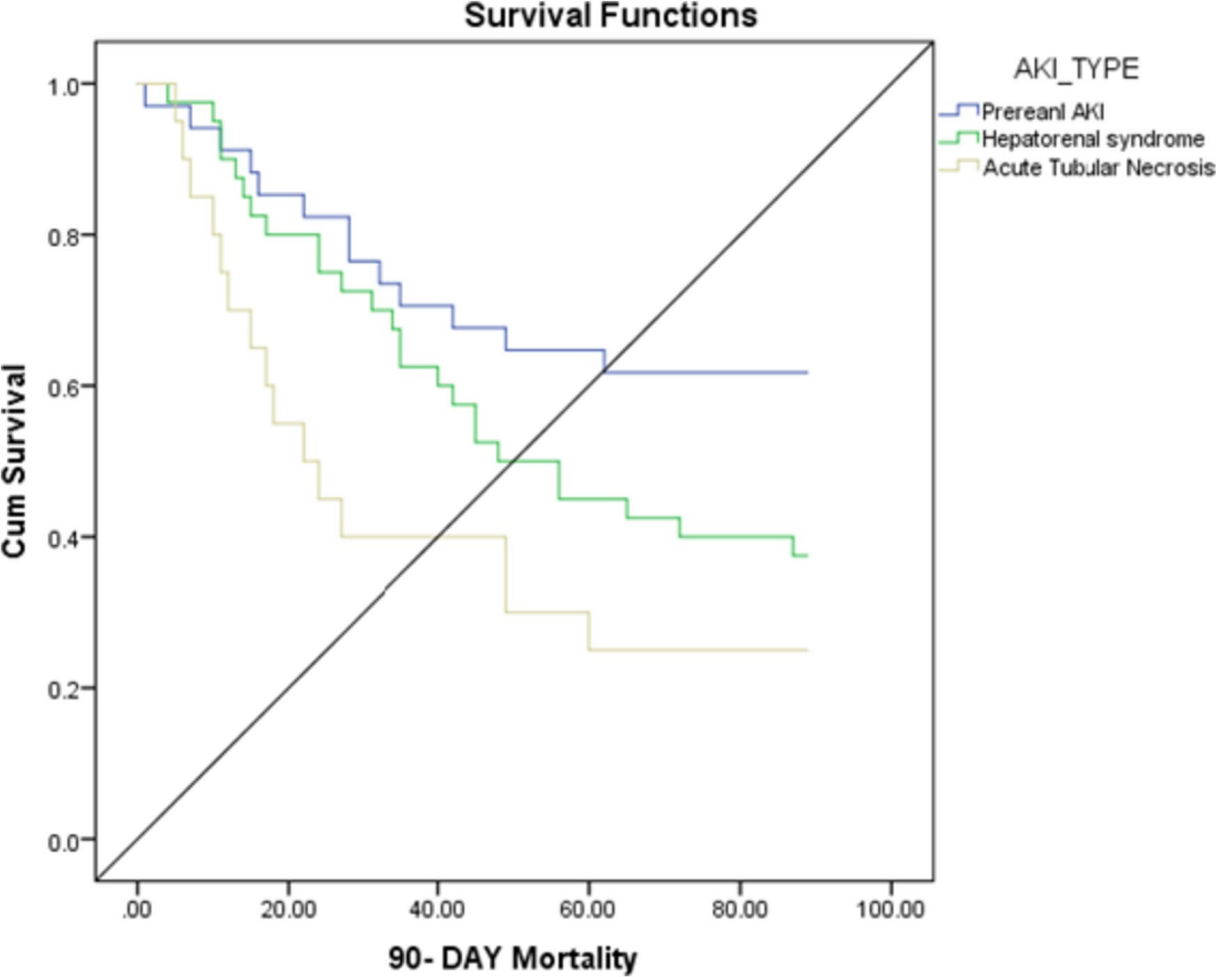
Kaplan–Meier survival curve showing 90-day survival in AKI types

A 30-day and 90-day mortality was significantly higher in the ATN group, followed by HRS, respectively. Kaplan–Meier survival curve showed significantly (*p* value 0.010) higher mortality rate in the ATN group on day 90 (Table 2 and Fig. 2).

**Table 2:** A 30-day and 90-day mortality in AKI types

Variables	AKI type			*p* value
	Prerenal AKI	Hepatorenal syndrome	Acute tubular necrosis	
30-day mortality	8 (23.5%)	11 (27.5%)	12 (60.0%)	0.014*
90-day mortality	13 (38.2%)	25 (62.5%)	15 (75%)	0.018*

### Mortality and AKI severity

**Fig. 3:**
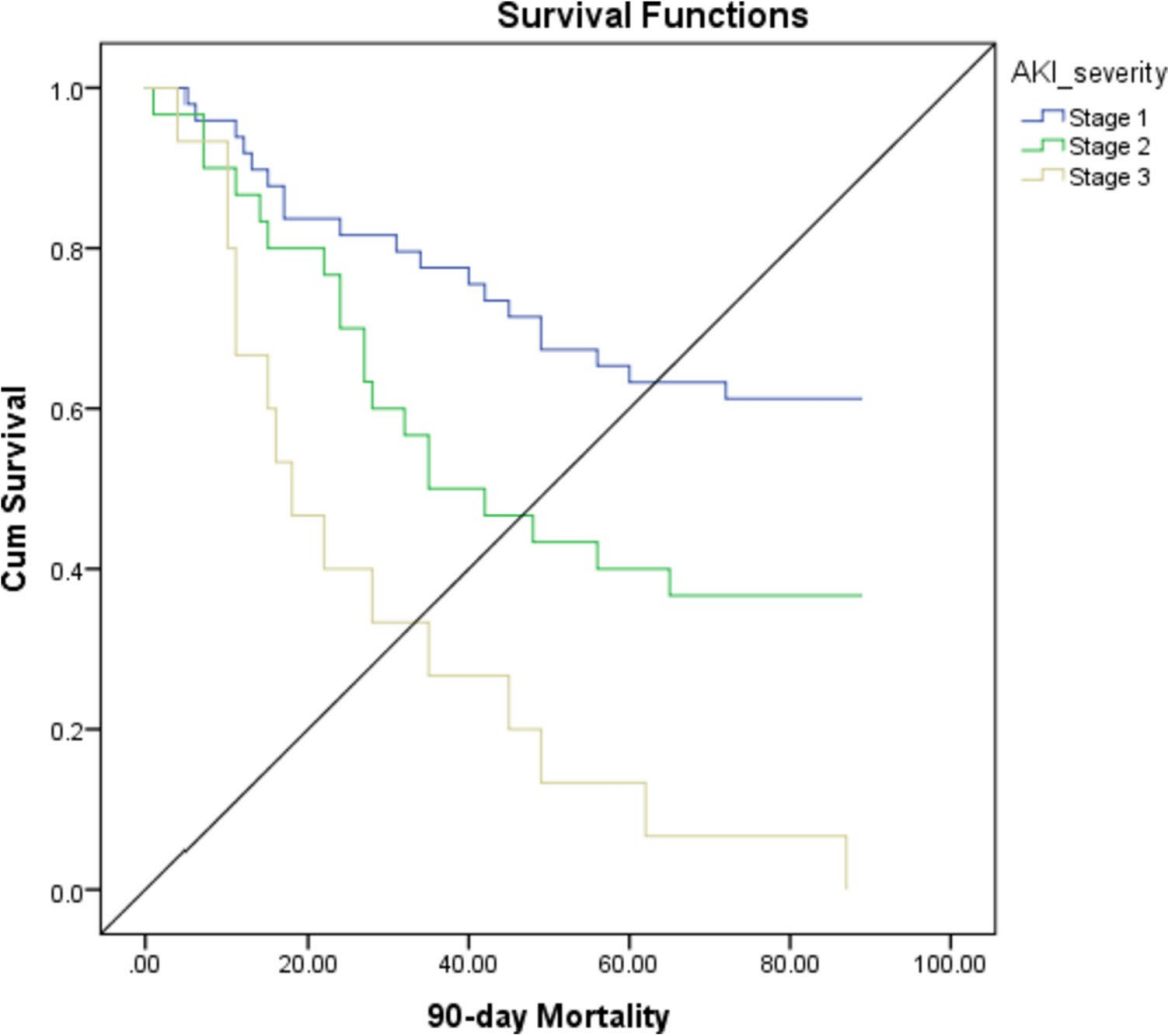
Kaplan–Meier survival curve showing 90-day survival in AKI staging

A 30-day and 90-day mortality was significantly higher in AKI stage 3 followed by AKI stage 2, respectively. Kaplan–Meier survival curve showed significant (*p* value 0.001) higher mortality in stage 3 followed by stage 2 on day 90 (Table 3 and Fig. 3).

**Table 3:** 30 days and 90 days mortality in AKI Severity Group

	AKI severity			*p* value
Variables	Stage 1	Stage 2	Stage 3	
30-day mortality	9 (18.4%)	12 (40%)	10 (66.7%)	< 0.05
90-day mortality	21 (44.6%)	17 (56.6%)	15 (100%)	< 0.05

## Discussion

The prevalence of AKI in this study was 25.2%, which is in agreement with the previously published literature [3, 20, 21]. A lower prevalence of 12.9% and 17.0% was reported by Choi et al. [1] and Terra et al. [22], respectively. Gessolo Lins et al. [23] reported a prevalence of 53.9% which was notably higher compared to the 25.2% prevalence observed in the present study.

The individuals involved in this study had a mean age of 51.46 years. The most common etiology was similar to that of national and international cohorts alcoholic cirrhosis [24], followed by NAFLD. A similar trend was observed in previous studies conducted by Thapa et al. [25] in Nepal where the mean age of their study groups was 51.8 years. In a study conducted by Gessolo Lins et al. [23], it was similarly noted that the primary cause of AKI in patients with liver cirrhosis is alcohol consumption.

When comparing the two groups, the analysis showed that individuals with AKI exhibited a notably higher prevalence of liver cirrhosis-related symptoms such as jaundice, edema, and icterus in comparison to the non-AKI group. Furthermore, laboratory investigation showed that total bilirubin, direct bilirubin, potassium, creatinine, urea, and INR was significantly higher among AKI patients than those without AKI (*p* < 0.05). Our data agreed with the study conducted by Metha et al. [26], Duah et al. [3], and Lasheen et al. [27], where laboratory parameters like bilirubin, potassium, creatinine, and INR were associated with AKI. In the present study, we observed significant decrease in serum albumin, globulin, and sodium (*p* < 0.05) which was in line with the study recently conducted by Duah et al. [3].

The regression analysis conducted to identify risk factors showed that there were differences in the renal risk profile between the cirrhosis and cirrhosis with AKI. Notably, higher levels of direct bilirubin and a higher MELD score emerged as significant risk factors associated with AKI development in liver cirrhosis patients. In a study by Tariq et al. [28], they reported that factors such as the MELD score, CTP stage C, the presence of ascites, and the presence of sepsis were predictors accompanying with AKI. Similarly, Gameiro et al. identified MELDNa as an independent predictive factor for AKI [29]. Another study also have also pointed to CTP, INR, total bilirubin, serum albumin, platelet count, total leukocyte count, presence of spontaneous bacterial peritonitis, and septic shock as the risk factors contributing to the AKI development [6]. A recently published systematic review and meta-analysis conducted by Nall et al. showed that high MELD score, infection, high CTP stage, high SCr, high serum bilirubin, and low serum albumin were significantly associated with a high incidence of AKI in liver cirrhosis patients [30]. All these studies corroborate our findings, emphasizing the parameters associated with a heightened risk of AKI in liver cirrhosis patients.

In our study, according to the updated 2015 ICA categorization, AKI stage 1 was the predominant stage observed, followed by stage 2 AKI. A similar trend was noted in the study by Thapa et al. [25], where the majority of the study population had stage 1 AKI (42%), followed by stage 3 AKI (30%). In a study by Huang et al. [31], among 217 patients with AKI, 132 (60.8%), 58 (26.7%), and 27 (12.4%) patients met ICA-AKI stages 1, 2, and 3, respectively. Regarding the etiological classification of AKI, the majority of cases in our study were prerenal AKI type. In study conducted by Moreau et al. [32], the most common causes of AKI in cirrhotic patients are PRA. Forty-nine percent of the patients had PRA, and 35% accounted for ATN.

AKI poses a heightened risk of mortality in many individuals with liver cirrhosis. Even patients with mild renal impairment (peak AKI stage 1) experienced significantly higher 90-day mortality rates compared to those without any renal impairment [2, 33]. In the present study, the over-all in-hospital mortality rate was 4.37%, while 30- and 90-day mortality rates were 14.01% and 23.35%, respectively, with high MELD score and presence of AKI being the independent risk factors for mortality in the study population. A study by Musunuri et al. [34] showed that INR and severity score (CTP) predict 90-day mortality in individuals with AKI in liver cirrhosis. A recent study conducted in Vietnam also found that hyponatremia, increased total bilirubin, and prothrombin < 70% substantially expanded the mortality percentage in patients with decompensated cirrhosis [35].

Among AKI classification, a 30-day (*p* = 0.014) and 90-day (*p* = 0.018) mortality rate was significantly higher in ATN group followed by HRS, respectively. Additionally, with respect to AKI severity, 30 days and 90 days mortality rate was significantly higher in AKI stage 3 followed by AKI stage 2, respectively. In a prospective study by Thapa et al., mortality rate was higher among patients with AKI stage-3 compared to AKI stage 1 and 2 [25]. Another study on 192 hospitalized liver cirrhosis patients reported that in-hospital mortality differed from 2% for AKI stage 1, 7% for AKI stage 2, and up to 21% for AKI stage 3. Additionally, the mortality rates for stage 1 patients who progressed to stages 2 and 3 were 29% and 60%, respectively [36]. Another study also revealed a substantial correlation between rising AKI severity and hospital mortality [37]. A study by Allegretti et al. reported that HRS and ATN result in similar 90-day mortality [38]. A recent study by Patidar et al. showed the lowest mortality rate in patients with PRA, while mortality were higher in ATN (52.7%) but not significantly different from HRS (49.0%) [39].

The Kaplan–Meier survival analysis showed that liver cirrhosis patients with AKI experienced the highest 90-day mortality rates, which aligned with the study conducted by Nguyen et al. [35]. Furthermore, there was an observation that the stage and type of AKI had an impact on patient survival rates. The present study indicated a statistically significant increase in mortality (*p* value 0.010) among patients in the ATN group and those at stage 3 on day 90.

Limitation of our study is its single-center design, which may restrict the generalizability of our findings. Additionally, the relatively small sample size utilized in this study could potentially limit the statistical power and precision of our results. In spite of these limitations, a significant contribution of this study is its emphasis on the association between AKI and heightened mortality rates among hospitalized individuals with cirrhosis. The findings of the correlation between survival rates and the type and stage of AKI contributes to the need for enhanced management strategies for the betterment of patient outcomes.

## Conclusion

In conclusion, this study underscores the frequent occurrence of AKI as a complication in individuals with liver cirrhosis, underscoring its significant association with short-term mortality rates. Elevated levels of direct bilirubin and MELD score were identified as notable factors linked to AKI development. Moreover, AKI and MELD scores emerged as independent risk factors for mortality, with notably higher rates observed at 30 and 90 days. Survival analysis demonstrated notably higher mortality rates on day 90 in AKI stage 3 compared to stage 2, as well as in cases of ATN compared to PRA. These findings stress the critical importance of early detection and management of AKI in liver cirrhosis patients to mitigate associated mortality risks. It is imperative to identify independent risk factors associated with AKI development to promptly recognize and intervene in this susceptible population.

## Data Availability

Data will be provided by corresponding author upon request.

## Abbreviations

AKI: Acute kidney injury
AST: Aspartate aminotransferase
ATN: Acute tubular necrosis
CTP: Child–Turcotte–Pugh
HRS: Hepatorenal syndrome
ICA: International Club of Ascites
MELD: Model for End-stage Liver Disease
NAFLD: Non-alcoholic fatty liver disease
PRA: Prerenal AKI
